# Sensory Evaluation of Pralines Containing Different Honey Products

**DOI:** 10.3390/s100907913

**Published:** 2010-08-26

**Authors:** Jovanka V. Popov-Raljić, Jovanka G. Laličić-Petronijević, Aneta S. Georgijev, Vladimir S. Popov, Mića A. Mladenović

**Affiliations:** 1 University of Belgrade, Faculty of Agriculture, Belgrade, Nemanjina 6, Serbia; E-Mails: jpopov@agrif.bg.ac.rs (J.V.P.R.); pcela@agrif.bg.ac.rs (M.A.M.); 2 “Timomed” d.o.o., Veljka Vlahovića b.b., Knjaževac, Serbia; E-Mail: timomed@nadlanu.com (A.S.G.); 3 University of Novi Sad, Faculty of Agriculture, Trg D. Obradovića 8, Serbia; E-Mail: vladapopov78@gmail.com (V.S.P.)

**Keywords:** dark chocolate pralines, *Apis mellifera carnica Poll* drone larvae, blossom honey, pollen, sensory attributes, storage

## Abstract

In this study, pralines manufactured by hand were evaluated sensorially. These pralines were obtained from dark chocolate containing 60% cocoa components, filled with *Apis mellifera carnica Poll* drone larvae, blossom honey and a blossom honey/pollen mixture from the protected region of Stara Planina-Eastern Serbia (a specific botanical region). The objectives of this study were investigations related to the use of sensory analysis for quality assessment of new functional products with potential benefits for human health, in particular of desserts based on dark chocolate pralines filled with different bee products characterized by a specific botanical and geographic origin, as well as of their storage properties and expected shelf life. Sensory quality (appearance, texture, odor and taste were evaluated by a group of experienced panelists immediately after the production (day 0), and then after 30, 90 and 180 days of storage under ambient conditions (temperature 18–20 °C). The results were statistically analyzed by the two-factorial analysis of variance (MANOVA) and with the LSD-test. It is possible to conclude that the storage time and composition of dark chocolate pralines containing different honey-bee products have statistically highly significant (p < 0.01) influence on the sensorially evaluated properties of pralines.

## 1. Introduction

Recently, dark chocolates produced with higher proportions of cocoa (at least 40 percent of the dry substance of cocoa components), are attracting considerable scientific and public interest, compared with milk chocolates, because dark chocolates contain increased amounts of tannic substances [1]. The latest investigations showed that tannic substances in a broader sense, wich includes condensation products of *o*-catechin, flavin diols and -triols, flavonoids, anthocyanidines and anthocyanins, besides the fact that their proportions determine the color and flavor of cocoa-beans [2], they also decrease the migration of dangerous substances through the cell membranes of human organisms, and their phenol residues act as natural antioxidants that are active in the metabolism [3]. Flavin triols are identified as the main substances responsible for the antioxidant properties of chocolate [4].

Cocoa-beans, as basic raw material for dark chocolate production, as well as cocoa-products, contain polyphenols that express similar, or even higher antioxidant capacities than those obtainable from some kinds of fruits or vegetables. Cocoa polyphenols can be divided into three groups of substances: flavin-3-ols (37 percent), anthocyanins (4 percent) and proanthocyanidines (58 percent). In cocoa-products (−)-epicatechin, (+)-catechin, traces of (+)-gallocatechins, (−)-epigallocatechin, (−)-epicatechin-3-gallate, numerous proanthocyanidines and small quantities of quercitin, quercitin glycoside, naringenin, lutheolin, apigenin, clovamide and phenolic acids, such as caffeic acid, gallic acid and *p*-coumaric acid, were identified [5].

In different research reports cocoa polyphenols were treated as bioactive components with antioxidative, antiradical and anticancerogenic properties [6,7]. Concretely, the forms of anthocyanogins isolated from cocoa have biological activity that is potentially significant for the protection of organisms from antioxidases and for the immune system.

Chocolate products share consumers' preferences with other confectionery products, especially due to their chemical and physical properties perceived by consumers as unique sensory attributes. Molded chocolates such as pralines are quite popular and can contain a center filled with fruits, creams, liquors, *etc*. These combinations have a significant effect on the products' texture and taste, and thus on its acceptability. Ziegleder *et al*. [8] analysed factors that effect the quality and stability of pralines with nougat filling and filling obtained with milk fat and fractionated palm-seed oil, during their storage for up to 500 days at 5–23 °C, while Rohm *et al*. [9] published their results concerning the application of the so-called one-shot praline production technique, with the simultaneous dosing of the filling, and of chocolate.

Recently performed investigations [10] point to the technique of forming pralines according to the triple-shot principle, which enables the simultaneous incorporation of three viscous liquids, with the application of a barrier layer (palm-seed oil alone or in the combination with milk fat) that prevents surface bloom of chocolates. As a disperse phase, pulverized sugar or skimmed milk powder were used.

The most comprehensive inquiries into the migration of fatty substances from praline filling (nougat filling, filling containing partially hydrogenised coconut oil or filling containing a mixture of milk fats) and polymorphous transformations of cocoa butter, are found in the 2006 study of Ziegleder *et al*. [11]. The praline samples were stored for the different periods of time and subjected to analysis by DSC (differential scanning calorimetry). The obtained DSC curves showed the migration of the liquid oils from different fillings into the chocolate jacket. Polymorphous transformations of cocoa butter crystals were only observed in the pralines with nougat filling, which were characterized by transitions from the β(V) in β(VI) form, and as a result, showed fast bloom development.

In a recent investigation of the functional foodstuffs market in the USA, it was stated that approximately 63 percent of population consumes functional foodstuffs as part of their nutrition, and that 94 percent of consumers believes that the intake of such foodstuffs can reduce risks of occurrence of different diseases, especially of cardiovascular diseases [12]. Honey and other bee products (pollen, propolis, royal jelly...) are considered as such foods, widely respected for their high nutritive value and protective characteristics [13,14]. Honey is sweet substance produced by *Apis mellifera* bees from the nectar of plants or from secretions of living parts of plants or excretions of plant-sucking insects on the living parts of plants, which bees collect, transform by combining with specific substances of their own, deposit, dehydrate, store and leave in honeycombs to ripen and mature [15].

Due to its sweetness, color and flavor, honey is often used as a replacement for sugar, as a component, or as a natural protective agent, in a lot of industrially produced foodstuffs [13]. Its composition depends on the kind of flowers (plants) from which honeybees collect the nectars, and other factors (such as environmental conditions and climate, for example). Besides sugars, honey contains numerous other components, of which many, including the polyphenols, have the antioxidative properties [13,16–19].

Whereas the average intake of sweeteners per person is estimated to be more than 70 kg per year, replacements of the traditional sweeteners in some foodstuffs with honey can result in improved antioxidative defense for healthy people [14,16].

Besides honey, it is important to point out some quality properties of pollen and its composition regarding content of bioactive components [14]. The chemical composition of pollen varies with plant species, environment during pollen development, age of plant when pollen developed, nutrient status of the plant, methods of pollen extraction, and storage [20–22]. The content of macro- and micronutrients of pollen has been published in many papers, and the influence of the botanical origin is indisputable [23,24].

Sensory properties of pollen also vary depending on its botanical origin. Thus, color varies from white to black, mostly being yellow, orange or yellowish-brown, but many different colors are possible according to the floral sources. Appearance of pollen is in the form of heterogeneous grains with different shapes and sizes, but spherical ones predominate. Odor is typical for pollen, specific for each flower source, and the taste intrinsic, sweet, sour, bitter or full [25].

Pollen grains are best preserved mixed-in with honey, in dark and hermetically closed packaging. The shelf life of fresh pollen is about one year, and that admixtured into honey two years, from the date of separation the pollen from the melliferous bee [26].

In some parts of Africa, Asia and Latin America, there is special economic interest in the collection and growth of definite species of insects, such as the larvae of melliferous bee drones, for use in the nutrition of the inhabitants. In many cultures insects are traditional foodstuffs, because of their high content of the nutritive substances, especially of proteins, and for their taste and specific characteristics, they are considered delicacies [27,28].

Investigating the nutritive composition of melliferous bee drone nest and its potential as a food for humans, Finke [29] stated that larvae and cocoons have high contents of proteins, fats and carbohydrates. Though their calcium content is low, bee offspring are a good phosphorus, magnesium, potassium, iron, zinc, copper and selenium source. They also contain essential amino acids, as well as the vitamins of B-group, vitamin C and choline. In summary, it is already recognized that insects, and among them melliferous bee (larvae and cocoons) can be the good sources of proteins [30–32]. As it was shown, they contain high quantities of proteins [33] of good quality [34,35].

It should be emphasized that, according to the definition, a praline is a product for one chew, which is composed of the chocolate corpus (at least 25 percent of the total weight), filled with a nougat mass, jelly, hazelnuts, marzipan or other fillings [36–39]. In Serbia, pralinea are most often considered to be a chocolate dessert filled only with the fondant filling. To the best of our knowledge, honey pralines cannot be found on the market in Serbia, neither as domestic nor as imported products, so that they should be considered as potentially new products of the confectionary industry, with, possible, long-lasting shelf life.

Considering all above mentioned facts, the aim of this paper was manual production of “functional” desserts—praline**s**—containing dark chocolate with 60% of cocoa solids as shells, filled with different honey bee products (honey bee drone larvae, blossom honey and blossom honey/pollen mixture) characterized by a specific botanical and geographic origin. Investigations were oriented towards one of relevant factors of overall quality, and that is—sensory analysis—employing a standard scoring method (appearance, texture and aroma).

## 2. Experimental Section

### 2.1. Materials

#### 2.1.1. Bee product collection areas

The first part of the experiments was performed at the apiary of the Centre for Selection and Reproduction of Queens of the “Timomed” company d.o.o. from Knjaževac—Serbia, where the blossom honey, pollen and the drone’s nests were collected.

The apiary is located on a mild slope approximately hundred meters from the Beli Timok river (The White Timok) at the foot of Stara Planina (the Old Mountain), which was, based on the proposal of the Institute for Nature Protection of Serbia, declared a Nature Park by the Government of the Republic of Serbia [40]. According to this Regulation, the mountain is juridically protected as a natural property with extraordinary significance, ranked as first category.

In the woods of Eastern Serbia, different woody plants can be found, such as hazel (*Corylus avellana*), dogwood (*Cornus mas*), elm (*Ulmus*), maple (*Acer campestre*), horse chestnut (*Aesculus hippocastanum*), acacia (*Robinia pseudoaccacia*), heart-like linden (*Tilia cordata*), sycamore (*Acer pseudoplatanus*), oak (*Quercus robur*), beech (*Fagus silvatica*), cerris (*Quercus cerris*), European ash (*Fraxinus*), white hawthorn (*Crataegus monogyna*), black hawthorn (*Crateaus nigra*), dog rose (*Rosa canina*), raspberry (*Rubus idaeus*), blackberry (*Rubus fruticosa*), blackthorn (*Prunus spinosa*), huckleberry (*Vaccinium myrtillus*), indigo bush (*Amorpha fruticosa*), walnut (*Juglans regia*), *etc*.

In the semi-mountainous regions large areas are occupied by natural meadows with many plant species. In the lower regions a number of artificial meadows and feed cultivation areas are present, with blooming occuring from about the middle of April until July. The significant herbaceous, melliferous plants include dandelion (*Taraxacum officinale*), coltsfoot (*Tussilagofarfara*), fragrant hellebore (*Helleborus odorus*), different species of clover (*Trifolium*), Bird’s foot trefoil (*Lotus corniculatus*), sainfoin (*Onobrychis viciaefolia*), common St. Johnwort (*Hypericum perforatum*), annual hedgenettle (*Stachys annua*) and others.

In the Timok valley large areas are occupied by different fruit cultures that enable fast development of bee colonies in the Spring. The most often found fruit species are plums (*Prunus domestica*), mirobalan plum (*Prunus cerasifera*), apricot (*Prunus armeniaca*), sweet cherry (*Prunus avium*), sour cherry (*Prunus cerasus*), apple tree (*Malus domestica*), pears (*Pyrus communis*), quince (*Cydonia oblonga*), raspberry (*Rubus ideaus*), blackberry (*Rubus frutiosa*), service tree (*Sorbus domestica*) and others [41].

#### 2.1.2. Bee products

The larvae of melliferous bee drones (*Apis mellifera carnica Poll*) were up to 10 days old, and they were collected together with comb. The basic composition of the raw larvae was: 32.02 percent of proteins; 25.41 percent of fats; 1.02 percent of cellulose; 3.53 percent of ash; and 3.46 percent of water. Larvae were frozen at −18 °C and, after defrosting at ambient temperature, they were expelled from combs with tap water pressure, after which they were thermally treated by roasting at about 100 °C for 3–5 minutes. After cooling, they were used as the dry larvae for the production of the filled dark chocolate pralines.

Blossom honey was collected at the apiary of the Centre for Selection and Reproduction of Queens of the “Timomed” company d.o.o.—Knjaževac, Serbia. A more detailed characterization of this honey was performed at Intertek Laboratories (Bremen, Germany). The analyzed sample contained 17.3 percent water, 11.3 mg/kg pH HMF, 19.3 m mol/kg of free acids, and its diastase activity was 23.4 DZ units. The analysis showed that the sample corresponds to the legal regulations [15].

The pollen, collected by bees (mainly during the morning hours, from 7 until 11 o’clock), was gathered with a pollen collector inside the beehive. The abovementioned laboratory found pollens in a pollen sample corresponding to the following botanical types: locust tree (*Gleditsia*) sunflower, dandelion, cockleburs (*Hellianthus, Taraxacum, Xanthium*), dogwood (*Cornaceae*), sedges (*Cyperaceae*), wild grass, corn (*Zea*), rosemary (*Labiatae*), alfalfa (*Lotus*), black lotus (*Robinia*), red clover (*Trilolium pratense*), white clover (*Trifolium repens*), meadowsweet (*Filipendula*), pears/plums (*Pyrus/Prunus*), willows (*Salix*), coriander (*Coriandrum*), grapevines (*Vitis*). According to this, the laboratory concluded that the honey may be called “blossom honey” from the“Eastern Europe” region [15].

Market globalization and the creation of conditions for the free exchange of commodities between countries and continents, have generated the necessity of identifying of the origins of goods. Consumers expect to have appropriate information on the quality and origin of goods that are marketed, *i.e.*, to have the opportunity of comparing products of similar kinds or categories based on their declared properties [15].

At that time, consumers give high significance to the products with a protected geographic origin, as the symbol of warranty of the specific quality. Products characterized by specific properties, owing to their quality, can be commercially successful. Honey can be considered as such product, owing to its relatively limited production and high prices. Because of that it is an substance prone to various adulterations and manipulations [42].

#### 2.1.3. Manual pralines production

The second part of the experiments was realized under laboratory conditions at the Faculty of Agriculture, University of Belgrade. For production of the praline shell, dark chocolate with 60 percent of cocoa components, containing 5.5% of proteins, 49.00% of carbohydrates and 35.00% of fats was used. Three types of samples were produced, according to the following list:

Sample 1: Dark chocolate praline enriched with honey-bee drone larvae;
Sample 2: Dark chocolate praline filled with blossom honey;
Sample 3: Dark chocolate praline filled with a mixture of blossom honey and pollen grains.

**Figure 1.**
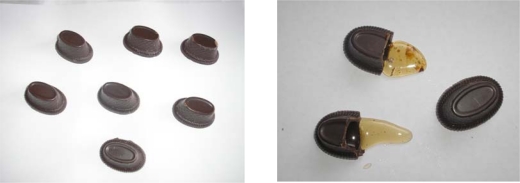
Visual appearance of hand made pralines with different bee-product fillings after 180 days of storage at ambient temperature.

The number of pralines manufactured was *n* = 100 for every examination period, in other words, in total, 400 pralines of each kind of filling were produced. The production procedure for pralines is relatively simple. The pre-prepared and tempered chocolate mass is manually poured into the selected forms (moulds) in a quantity necessary for obtaining walls of predetermined thickness (≈3 mm). The chocolate shell is then, together with the mould, cooled in a refrigerator, inducing chocolate solidification and the obtaining of the final praline appearance. After removing from the refrigerator, shells are separated from the moulds and filled-up with the desired bee products, using a needle-filler with a plastic bag. After filling, shells are closed by pouring-over with tempered liquid dark chocolate and, after cooling, praline production is finished. The net weight of one praline was 12 grams, of which 7 grams was the shell, and 5 grams the filling (Figure 1).

Manually produced praline quality was tested sensorially immediately after processing (day 0), and then after 30, 90 and 180 days of storage at the ambient temperature. Some of the factors that could lead to changes—deterioration of sensory quality of pralines, as well as their reduced stability, apropos shortening of their shelf life, were presented in the Introduction section. Generally the recommended shelf life for chocolate desserts like pralines is about three months, depending on the type of filling, while the sustainability of chocolate is up to one year. Since the pralines prepared for the purposes of this study were made with types of fillings that have long shelf lives, the authors have chosen as the period of storage up to 180 days, as it lies in the middle of previously mentioned periods of sustainability, and, also, in accordance with the criteria of Rhom et al. [9].

### 2.2. Sensory analysis

The sensory properties of the dark chocolate pralines were investigated during the whole period of storage, using the known relevant ISO standards [43–50], and the scoring procedure was identical to that applied for assessment of dietary chocolates in our previous work [51]. For the readers’ convenience, this scoring schedule is reproduced in Table 1.

*Quality category* was determined depending on score ranges; products which were evaluated with less than 2.5 points were considered as unsatisfactory, *i.e.*, as inacceptable; scores within the 2.5–3.5 range characterized good quality products, 3.5–4.5 very good quality and 4.5–5—excellent products.

Objective, accurate and reliable sensory analysis is possible only if terms and definitions that are given in the Standard ISO 5492:2000—Sensory analysis—Vocabulary [46] are well known. This Standard defines four groups of terms: general terms, terms related with senses, terms defining sensory qualities, and terms respecting the methods of immediate testing (methods of sensory analysis).

An important provision for the objectification of sensory analysis is the consistent application of the precisely defined procedures for selection of sensory assessors, their permanent training and systematic monitoring. Sensory analysis, as part of objective and reliable determination of the food product quality, can be performed only by selected, trained and verified persons, in accordance with ISO Standards 8586—Sensory analysis—General Guidance for the Selection, Training and Monitoring of Assessors—Part 1 (1993): Selected Assessors and Part 2 (1994): Experts [44,45]. That is, for impartial and trustworthy sensory evaluation only selected assessors can be used, especially expert assessors and specialized expert assessors.

The method of scoring (evaluation) includes selection of the representative quality attributes that are going to be evaluated by specialized expert assessors for the particular foodstuff, which is being investigated. Irrespectively to the number of scores, *i.e.*, of span of scores, each quality level—expressed with the corresponding score (point), has to be precisely defined. In the majority of cases, it is adequate to apply score list with 5 points (1 to 5), as long as no so-called “dead” scores, appear, *i.e.*, when the quality levels that were not precisely defined. Differences between scores must not be too great, but they have to be high enough, so that the assessors could distinguish them [52].

Besides the indisputable fact that for the scoring method attributes (appearance, texture, flavor) that are representative for the quality of the distinctive foodstuff have to be chosen, it is clear that each of those characteristics does not have equal effects with respect to the total quality of the examined product. Having this in mind, Planck, as far back as in 1947, introduced and scientifically explained the so-called “weight coefficient” (according to the statements quoted in [53]). By application of weight coefficients, a quantitative expression of the total product quality is obtained as the “weighted” mean value of the scores for each the evaluated parameter. Because of that, before performing the evaluations, it is important to determine the weight coefficient for each property, and balance them in such a way, that their sum equals 20.

In order to objectify and ensure the reliability of sensory analysis it is necessary to perform the correct procedures of selection, training and verification of the assessors (sensory analysts). In addition to the assessors and sensory analysts, for the specific situations of quality evaluation of food products (obtaining expert’s opinions), the specialized expert assessors category has remarkable importance. The problems related with this category of assessors is regulated by the special international standard ISO 8586-2: 1996: Sensory analysis—General Guidance for Selection, Training and Monitoring of Assessors—Part 2: Experts [45]. An expert assessor is any person, who, based on his/her knowledge and/or experiences, as well as on their verified competency, is chosen to elaborate an opinion in the domain for which he/sheis competent. In the framework of this category, the Standard differentiates between two types of the assessors: expert assessors and specialized expert assessors.

Several methods exist for the selection, training and monitoring of assessors, but one of the most relatively simple methods is the ranking method [54]. The objective of the test is that for each individual assessor a so-called sensory sensitivity coefficient (S) is determined. The minimum value of this indicator has to be 1.0. For verification of the evaluating committee (panel) the method of circular experiments is used. Then an index of reproducibility (IR) is calculated according to the following equation:

(1) $$\mathrm{IR}=1+\frac{\sum_{i=1}^{n}\sum_{j=1}^{m}{\left(x_{\mathrm{ij}1}-x_{\mathrm{ij}2}\right)}^{2}}{n\cdot m}$$

*IR*—index of reproducibility
*x*_*ij*1_—number of points for the first assessment of product “i” for property “j”
*x*_*ij*2_—number of points for the second assessment of product “i” for property “j”
*n*—number of products that are evaluated twice
*m*—number of properties.

For a homogeneous evaluating committee (panel) the index of reproducibility (IR) varies in the range of 1.1 to 2.0, but it is considered that the optimum value should be to 1.35 [55]. The panel selected for the evaluation of pralines filled with different bee products consisted of 10 experienced specialized expert assessors for sensory evaluation of food products, including chocolates. Panelists all met the criteria specified by the ISO standards for selection, training and monitoring of assessors [44,45].

### 2.3. Statistical analysis

Data obtained in the investigations performed in this study were analyzed by descriptive and analytical statistics. Basic parameters of the descriptive statistics included calculations of the arithmetic mean values, and variability parameters of the investigated properties included determinations of standard deviations (Sd) and variation coefficients (Cv) expressed in percents.

For analytical statistics (evaluation of sensory determinations data of the chocolates), the two-factorial analysis of variance MANOVA was applied, with the first factor being the storage time, and the second one—the composition of the evaluated chocolate samples, as well as the LSD-test (test of the least significant differences of pairs).

For finding out if the prerequisites for variance analysis methods are justified, homogeneities of variances were determined using Levene’s test. For the data which, based on Levene’s test, for variances were homogenous, parameter statistics was applied, and in the case of inhomogeneity of variances, the parameter statistics was applied as well because in the employed two-factorial experiment it was not possible to apply the nonparametric statistics [Statistics—V.6-package] [56,57].

Within the scope of the instrumental determinations of characteristics of color measurement, the trend equation that was best adapted to the average reflectance changes (Y, %) was obtained with the statistical software Origin 6.1 (Origin Lab. Corporation, Northampton, MA 01060, USA).

## 3. Results and Discussion

### 3.1. Sensory evaluations of pralines produced with dark chocolate

Chocolates, as well as chocolate desserts of the praline type filled with different fillings or products, are regarded as popular delicacies by a broad range of consumers (healthy adults, children…). They have high energetic value (about 2,300 kJ/100 g) and consumption of moderate quantities of chocolate products induces an increased feeling of pleasure, as well the intake of biologically active substances that are useful for human health [3,5,58,59].

In consonance with the contemporary trends in production of foodstuffs, ever more investigations deal with the production of foods with specific functional properties, which provide particular benefits with respect to human health.The energetic value of chocolate and chocolate products is relatively high, and it can be decreased by using two approaches. The first of them is the reduction of fat content, but if the fat content is too low (bellow 27 percent), chocolate loses its smoothness and the inimitable sense of its slow melting in the mouth cavity disappears. The second solution is the replacement of sugars (sucrose) with sugar supplement sweeteners, which contribute to the corresponding physico-chemical and sensory properties. Polyols, such as isomalt, fulfill these requirements. Besides the fact that they have lower energetic value compared with that of the sucrose, they can be used by the specific groups of consumers, for example by diabetics, while they do not contribute to increasing the glucose levels in blood [60].

Despite all the abovementioned facts, sensory properties represent the most important parameters of chocolates and chocolate products, as consumers consume chocolate *exclusively as a product for sensory satisfaction (taste, odor, flavor),* and not as a general purpose foodstuff. From the consumer’s point of view, chocolate and chocolate products must have attractive appearance (color, surface brightness, form), corresponding firmness at ambient temperature, regular break, unique flavor—taste and odor, and satisfactory shelf life during storage and distribution. the sensory properties (appearance, color, flavor—odor and taste) of chocolate and chocolate products, other cocoa products and chocolate-like products and cocoa-based spread-products must be characteristic *i.e.*, specific for the declared product [39].

**Figure 2.**
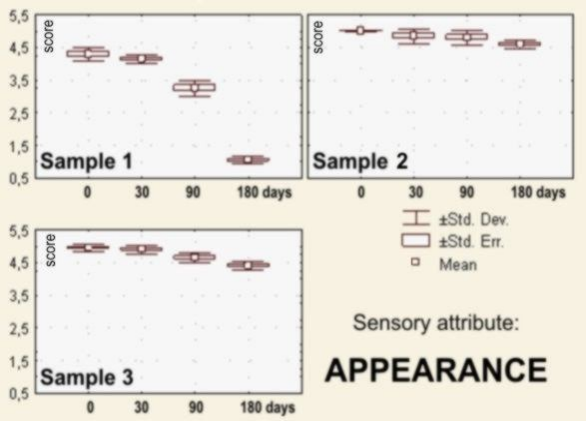
Box-plots for sensory attribute *appearance* of the filled pralines: **Sample 1**—filled with honeybee drones larvae; **Sample 2**—filled with the blossom honey; **Sample 3**—filled with combination of blossom honey and pollen, during 180 days of storage.

**Figure 3.**
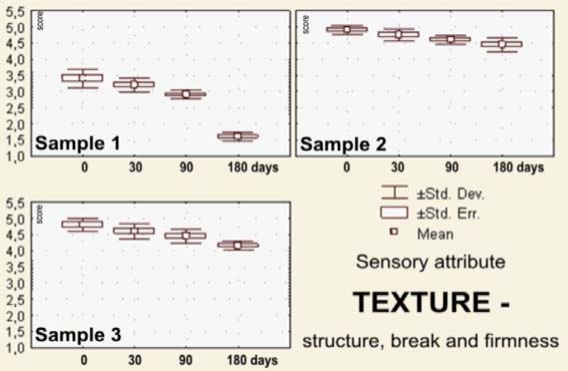
Box-plots for sensory attribute *texture*—structure, break and firmness of the filled pralines (labels as in the Figure 2), during 180 days of storage.

**Figure 4.**
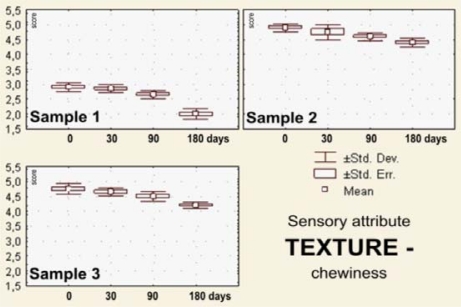
Box-plots for sensory attribute *texture*—chewiness of the filled pralines (labels as in the Figure 2), during 180 days of storage.

**Figure 5.**
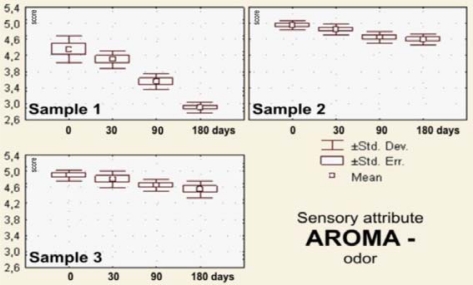
Box plots for sensory attribute *aroma—*odor of the filled pralines (labels as in the Figure 2), during 180 days of storage.

**Figure 6.**
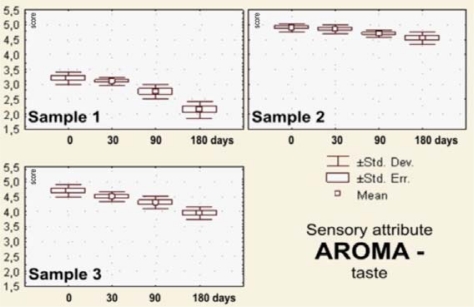
Box plots for sensory attribute *aroma—*taste of the filled pralines (labels as in the Figure 2), during 180 days of storage.

**Figure 7.**
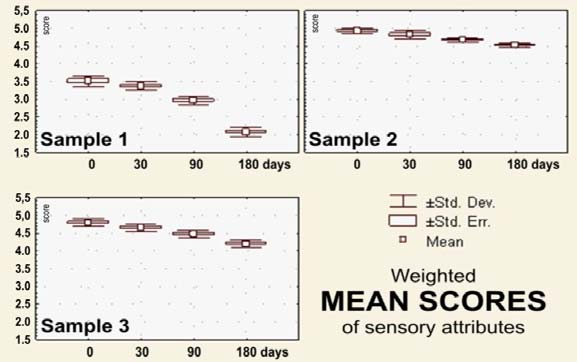
Box plots for *weighted mean score* for the evaluated sensory attributes of the filled pralines (labels as in the Figure 2), during 180 days of storage.

The sensory evaluation results the dark pralines filled with drone larvae (sample 1), blossom honey (sample 2) and a combination of blossom honey and pollen (sample 3) that were stored for 0–180 days at 18–20 °C are shown in Figures 2–7.

The visual impressions (appearance of the foodstuffs) are registered with the sense of sight (eyes) and they include color, shape, surface, structure...[61]. For the objectification of the sensory characteristics of color of food products, as well as of chocolate, it is recommended at the same time to perform instrumental determinations of the color quality parameters and to correlate the thus obtained results with the sensory impressions for nuance (hue), brightness and saturation of color [51].

Dark chocolate praline filled with the blossom honey (sample 2), immediately after the processing show the best appearance (appropriate dark-brown, *i.e.*, black color, the corresponding shape and surface brightness), so that they were evaluated with the highest score *x_m_* = 5.00. Immediately after the production, the score for the appearance of the sample 3 was a little bit lower *x_m_* = 4.95; S_d_ = 0.11; and C_v_ = 2.26, followed by sample 1 (*x_m_* = 4.30; S_d_ = 0.21; and C_v_ = 4.86). The appearance of samples 1 changed insignificantly after 90 days of storage (*x_m_* = 3.25; S_d_ = 0.25; and C_v_ = 7.69), and then quickly dropped to almost unacceptable values (*x_m_* = 1.05; S_d_ = 0.11; and C_v_ = 10.65), showing surface fat bloom, surface roughness and a lack of brilliance.

Appearance, especially surface smoothness and brilliance, represent the key factors of dark chocolate quality. The most often, and finally devaluating parameter of the appearance is so called fat bloom: the formerly brilliant chocolate surface becomes cloudy, and then acquires a grayish-white nuance [62]. The mechanism of formation of fat bloom is connected to the transformation of the thermodynamically unstable β(V) polymorph of cocoa butter into the more stable β(VI) polymorph [63]. This transformation can be induced by different factors (tempering, forming, cooling or inappropriate long-lasting storage [51,58,64–70]).

In the scientific literature, mixing other types of fat with cocoa butter, which leads to migration of fat from the interior to the surface of chocolate, is cited as one of the causes of fat bloom in chocolate [3,58,54]. The mentioned phenomena is particularly interesting in the case of filled chocolates, as well as praline-like products. Since the honey-bee drone larvae, used as a filling in sample 1, contained significant amounts of fat (25.41%), it is possible that the interaction between this fat and cocoa butter from the chocolate shell led to a faster decline in quality, especially appearance, because of bloom development.

Contrary to sample 1, samples filled with blossom honey and a combination of blossom honey and pollen (samples 2 and 3) maintained their appropriate—excellent appearance (color, brilliance, shape and surface) during the whole storage period (180 days; Figure 2).

Chocolate has a unique taste, aroma and texture, because of which it represent a favorite dessert for a great number of people. During its consumption, it melts at the temperature of the human body. In the mouth, the firm structure transforms into a thick suspension. These attributes and the physico-chemical properties, including the rheological properties, significantly depend on the technology used in the chocolate manufacturing procedure [60]. During the storage period of up to 180 days, the *textures* of the chocolate pralines from samples 1–3 were visually estimated as the break and palpatory structure estimated for the firmness of the chocolate shells. The best scores were ascribed to the praline samples filled with blossom honey (sample 2) and then to samples filled with blossom honey and pollen combination (samples 3). Pralines filled with honeybee drone larvae (samples 1) were scored considerably lower. Concretely, samples 1 were after 180 days of storage sensorially judged as unacceptable (*x_m_* = 1.60; S_d_ = 0.14; and C_v_ = 8.56) (Figure 3).

The remaining textural properties included estimations of chewiness and stickiness using the oral technique. Chewiness is one of the mechanical texture properties reflecting the cohesivity and time or number of chews that are necessary to chew a firm product into a shape ready for swallowing. For the praline samples filled with the honey bee drones, the chewiness property was scored with the lowest marks, irrespectively if it was for day 0 (*x_m_* = 2.90; S_d_ = 0.14; and C_v_ = 4.72), or for days 30 (*x_m_* = 2.85; S_d_ = 0.14; and C_v_ = 4.89), 90 (*x_m_* = 2.70; S_d_ = 0.11; and C_v_ = 4.14), or 180 (*x_m_* = 2.00; S_d_ = 0.18; and C_v_ = 8.84), being, on day 180, tough and sticky to the teeth. Opposite to sample 1, samples 2 and 3 preserved excellent swallowing properties until day 90, and then were very good until day 180 (Figure 4).

After the visual impressions, in the procedure of sensory analysis of chocolate products the odorous or olfactory sensations are applied. The sense of smell characterizes common sense information for recognizing odor quality, and at the same time the taste quality, which is of the primary importance for the evaluation of aroma (flavor) [71].

Chocolate products contain specific odors and tastes that are encircled with the additional flavor [3]. According to the statements of Neua quoted in [3], chocolate flavor is based on more than 400 compounds. Amino acids such as valine, leucine and phenylalanine are included in formation of phenyl acetaldehyde, which is significant for the flavor formation, being, probably, the consequence of the dark chocolate with a higher share of the cocoa components, as well as the consequence of chemical, *i.e.*, of the botanical and geographic origin (Eastern Serbia) of the blossom honey and pollen [41].

Values of the average scores for the olfactory estimated odors and orally estimated tastes of the analyzed praline samples (1, 2 and 3) are illustrated in the box-plots shown in Figures 4 and 5. Immediately after processing and after 30 days of storage of the samples filled with the blossom honey or the combination of blossom honey with pollen, odor and taste are appropriate, *i.e.*, of excellent quality, while after 90 and 180 days they were scored with insignificantly lower average marks. However, neither the sensory parameter odor, nor the sensory property taste, were scored with the maximum marks. The explanation can possibly be found in the sweetness of pralines filled with the blossom honey (sample 2) and the appearance of mild bitterness (sample 3). Taste and flavor are the two most important characteristics of honey. The sweet taste is the result of presence of sugars, constituting about 80% of honey, but also of the presence of sour and bitter notes. Aroma is the consequence of the complex mixture of volatile components, which varies as a function of the nectar origin, conditions of production and of storage [72,73].

The samples of pralines filled with larvae of honeybee drone rank (sample 1) for *taste,* as well as for *odor,* were characterized by an atypical odor and brackish taste (Figures 5 and 6).

The weighted mean value of scores for pralines made from dark chocolate filled with melliferous bee drone larvae was *x_m_* = 3.51, or 70.2% compared with the maximal possible quality. After 30 days of storage, that value decreased insignificantly, being 3.37, or 67.4% of the maximum possible quality. Storage for 90 or 180 days results in substantial changes, primarily in the appearance (fat bloom on the chocolate surface, missing brilliant and smooth surfaces), strange structure and break, as well as the firmness, and pronounced toughness and adhesion to teeth, with the deviation, *i.e.*, appearance of a nontypical flavor (*x_m_* = 2.08, or 41.6% compared with the maximal possible quality, Figure 7). This is probably accompanied by degradation or transformation of products, because the composition of raw melliferous bee drone larvae contains a significant amount of fat.

Bearing in mind that larvatic biomass is an animal product rich in crude protein and fat contents, it is likely to have a limited shelf life, which would primarily affect the sensory properties of these products. This is the main reason why we examined these kind of pralines during the reasonably long keeping period.

Praline filled with blossom honey (sample 2) retained excellent total sensory quality (appearance, texture and flavor) during the entire period of storage up to 180 days, with values of the weighted mean score immediately after production of *x_m_* = 4.92, or 98.4% compared with the maximal possible quality and *x_m_* = 4.52, or 90.4% after 180 days of storage (Figure 7). Dark chocolate pralines filled with the combination of the blossom honey and pollen were judged as excellent immediately after the production and after 30 days of storage (95.8 and 92.4% of the maximum quality, respectively). After 90 days, with *x_m_* = 4.47, or 89.3%, and after 180 days of storage, with *x_m_* = 4.19, or 83.94% (Figure 7) they could be considered as products with very good sensory quality.

The results of Levene’s test for homogeneity of variances for the praline samples filled with different bee products are outlined in Table 2. Based on these results it can be concluded that the data are homogenous for all evaluated sensory quality properties, with exception of the property of appearance, for which the variances were pronouncedly inhomogeneous (*F* = 2.881 and *p* = 0.005) and a property of the aroma—odor, where variances were inhomogeneous (*F* = 2.246 and *p* = 0.027).

On the basis of the two-factorial analysis of variances of the praline samples with filings from different bee products (Table 3), it is possible to state that the both investigated factors—time of storage and composition (filling) have statistically very significant influence (*p* < 0.01) on the investigated sensory quality properties. With respect to the interactions of the mentioned factors, it can be seen that for sensory property of texture—*chewiness* the interaction appears to be statistically significant, while for other sensory properties they appear to be statistically very significant (*p* < 0.01).

The appearance of the evaluated praline samples after up to 30 days of storage was not changed significantly, while after 90 days of storage that attribute changes statistically very significantly (*p* < 0.01).

When considering the composition of pralines (kind of filling), it is seen that between the pralines filled with the blossom honey and those filled with the combination of blossom honey and pollen there is no statistically significant difference. On the other hand, there is statistically a very significant difference with respect to the appearance (*p* < 0.01) between pralines with the mentioned fillings (samples 2 and 3) and those filled with melliferous bee drone larvae. Like the attribute of appearance, the sensory attribute of texture (structure) and of the chewiness for the analyzed samples does not statistically change significantly for up to 30 days of storage, but, after that period of until the end of the experiments a statistically very significant change of that attribute appears. With respect to the composition (kind of filling), there appear to be very statistically significant differences present (*p* < 0,01) for the attribute of chewiness between all analyzed samples of pralines (samples 1, 2 and 3).

Sensory attribute of aroma—odor after 30 days of storage statistically changes significantly, and after 90 or 180 days of storage that change was at the level of significance of *p* < 0.01. Pralines filled with blossom honey (sample 2) and those filled with a combination of blossom honey and pollen (sample 3) were not statistically different with respect to odor, but in comparison to the samples filled with the melliferous bee drone larvae (sample 1), their odor was statistically very significantly different (*p* < 0.01).

The change of the attribute of aroma—*taste* of the praline samples was statistically very significantly changed (*p* < 0.01) for the storage time longer than 30 days, but, before that period, changes of taste were not significant.

Based on the result of the LSD test, depending on the composition, *i.e.*, on the kind of filling, it can be concluded that the fillings of praline with blossom honey, or with combination of the blossom honey and pollen, or with melliferous bee drone larvae, induce very significant changes in taste (*p* < 0.01). Application of the LSD test showed that among the investigated samples of pralines with fillings of different bee products there are significant differences with respect to the weighted score for the factor *time of storage* in the dependence of the factor *composition (kind of filling)*.

## 4. Conclusions

- Pralines were produced manually from the dark chocolate with different bee products (*Apis mellifera carnica Poll* drone larvae; blossom honey; and blossom honey combined with pollen grains) from the region of Stara Planina-Eastern Serbia that is legally protected as a natural region with the extraordinary significance that possibly enables the production of “functional products” having corresponding benefits with respect to human health.
- On the basis of two-factorial analysis—MANOVA, the composition (dark chocolate with 60% of cocoa parts, *Apis mellifera carnica Poll* drone larvae; blossom honey; and blossom honey/pollen) and storage time (0, 30, 90 or 180 days), as well as their interactions, have statistically very significant effects (*p* < 0.01) on the evaluated sensory attributes (appearance—form, color, brightness, surface, texture—structure, break, firmness, chewiness and other textural properties; aroma—odor and taste). Therefore, shares of all components that are to be included in different desserts based on black chocolate must be completely harmonized in order for them to achieve fully acceptable and balanced sensory quality.
- Pralines from dark chocolate (with 60% of cocoa parts) filled with blossom honey obtained in the region of Stara Planina-Eastern Serbia (the defined geographic and botanic origin) had excellent sensory quality (appearance, texture and aroma), during the entire period of storage up to 180 days with the value of the average weighted scores of *x_m_* = 4.92 or 98.4% of the highest possible quality immediately after the production, and *x_m_* = 4.52 or 90.4 % after 180 days of storage.

## Figures and Tables

**Table 1.** Sensory evaluation of the chocolate praline quality using the scoring procedure.

**Basic sensory properties**	**Score**	**Weight coefficient**	**Description of the evaluated property**

APPEARANCE Form, color, brightness, surface	5	2	Appropriate form; irreproachable color; smooth, bright surface; clear engraving
	4		Insignificant deviation of form; irreproachable color; smooth, bright surface; engraving less clear
	3		Deviations of form; lower quality color; fingerprints on the surface; air bubbles; engraving less clear
	2		More pronounced form deviations; partially white of gray surface; presence of cuttings
	1		Form distorted; surface gray or white; higher damages; bad engraving

TEXTURE Structure, break, firmness	5	3	Break straight, homogenous, fragile; structure homogenous; texture smooth; firmness appropriate
	4		Break uneven; structure homogenous; firmness appropriate
	3		Break uneven, air bubbles; firmness inappropriate; fat bloom appearance on the break
	2		Break uneven; texture roughly-granular; fat bloom on the break
	1		Crumbling; texture roughly granular; fat bloom
	
Chewiness and other textural properties	5	4	Appropriate chewiness; melting in the mouth
	4		Slower melting; good chewiness, spreadiness
	3		Average chewiness; spreadiness; weak sandiness
	2		Slow melting; sandiness; stickiness
	1		Slow melting; heavy sandiness; stickiness

AROMA Odor Taste	5	4	Appropriate; rounded; aromatic
	4		Appropriate poorer rounded; aromatic
	3		Appropriate; poor rounded; weakly aromatic
	2		Not appropriate; sourish; staled
	1		Foreign odor; sour; staled; mouldy

	5	7	Appropriate; rounded; aromatic
	4		Appropriate, less rounded; aromatic
	3		Poorly rounded; poorly aromatic
	2		Sourish; not rounded
	1		Foreign taste; sour; bitter

**Table 2.** Results of the Levene test for the homogeneity of variances of the praline samples with different fillings.

**Sensory quality attribute**		**Levene’s test**	
		**F**	**p**
Appearance		*2.881*	*0.005*
Texture	Structure, break and firmness	0.706	0.727
	Chewiness	0.637	0.789
Aroma	Odor	*2.246*	*0.027*
	Taste	0.830	0.612
Weighted mean value of scores		0.801	0.639

**Table 3.** Results of the analyses of variances for sensory attributes of the praline samples with different fillings.

**Sensory quality attribute**		**Storage time**		**Composition (kind of the bee products for filling)**		**Interaction**	
		***F***	***p***	***F***	***p***	***F***	***p***
Appearance		227.189	*0.000*	628.980	*0.000*	106.366	*0.000*
Texture	Structure, break and firmness	73.353	*0.000*	613.681	*0.000*	16.773	*0.015*
	Chewiness	49.042	*0.000*	198.478	*0.000*	2.958	*0.000*
Aroma	Odor	43.731	*0.000*	198.470	*0.000*	11.492	*0.000*
	Taste	38.874	*0.000*	553.000	*0.000*	3.414	*0.007*
Weighted mean value of the scores		156.226	*0.000*	1502.620	*0.000*	27.011	*0.000*
